# Global Cardiovascular Research Output, Citations, and Collaborations: A Time-Trend, Bibliometric Analysis (1999–2008)

**DOI:** 10.1371/journal.pone.0083440

**Published:** 2013-12-31

**Authors:** Mark D. Huffman, Abigail Baldridge, Gerald S. Bloomfield, Lisandro D. Colantonio, Poornima Prabhakaran, Vamadevan S. Ajay, Sarah Suh, Grant Lewison, Dorairaj Prabhakaran

**Affiliations:** 1 Centre for Chronic Disease Control, New Delhi, India; 2 Northwestern University Feinberg School of Medicine, Chicago, Illinois, United States of America; 3 Duke University Medical Center, Durham, North Carolina, United States of America; 4 University of Alabama-Birmingham School of Public Health, Birmingham, Alabama, United States of America; 5 Public Health Foundation of India, New Delhi, India; 6 King's College London, London, United Kingdom; Max Planck Society, Germany

## Abstract

**Introduction:**

Health research is one mechanism to improve population-level health and should generally match the health needs of populations. However, there have been limited data to assess the trends in national-level cardiovascular research output, even as cardiovascular disease [CVD] has become the leading cause of morbidity and mortality worldwide.

**Materials and Methods:**

We performed a time trends analysis of cardiovascular research publications (1999–2008) downloaded from Web of Knowledge using a iteratively-tested cardiovascular bibliometric filter with >90% precision and recall. We evaluated cardiovascular research publications, five-year running actual citation indices [ACIs], and degree of international collaboration measured through the ratio of the fractional count of addresses from one country against all addresses for each publication.

**Results and Discussion:**

Global cardiovascular publication volume increased from 40 661 publications in 1999 to 55 284 publications in 2008, which represents a 36% increase. The proportion of cardiovascular publications from high-income, Organization for Economic Cooperation and Development [OECD] countries declined from 93% to 84% of the total share over the study period. High-income, OECD countries generally had higher fractional counts, which suggest less international collaboration, than lower income countries from 1999–2008. There was an inverse relationship between cardiovascular publications and age-standardized CVD morbidity and mortality rates, but a direct, curvilinear relationship between cardiovascular publications and Human Development Index from 1999–2008.

**Conclusions:**

Cardiovascular health research output has increased substantially in the past decade, with a greater share of citations being published from low- and middle-income countries. However, low- and middle-income countries with the higher burdens of cardiovascular disease continue to have lower research output than high-income countries, and thus require targeted research investments to improve cardiovascular health.

## Introduction

Economic development has been associated with transitions in health, notably shifts from communicable diseases to non-communicable, chronic diseases [NCDs] including cardiovascular diseases [CVD] [1]. Health research is one mechanism to improve population-level health and should generally match the health needs of populations. Even though CVD is the leading cause of death and disability worldwide, there have been limited data to assess the trends in country-level cardiovascular research output [2], [3]. Bibliometrics, a research evaluation methods used to quantitatively evaluate scientific literature, can be used to measure trends in research productivity over time. Prior cardiovascular bibliometric research has been largely confined to high-income country settings from more than 20 years ago [4].

Our objective was to evaluate trends in global cardiovascular research publications disaggregated by country from 1999 to 2008, the relative importance of these publications as measured by average number of citations per paper, and the degree to which countries collaborate. An additional aim was to compare cardiovascular research publications against measures of country-level development and CVD burden.

## Methods

### Bibliometric Filter Creation and Testing

We created, tested, and revised a cardiovascular bibliometric filter using a previously published approach to achieve >90% precision (specificity) and recall (sensitivity) to capture cardiovascular research articles, reviews, and conference proceedings from the Web of Knowledge (1999–2008) [5], [6]. Precision represents proportion of filter output considered to be cardiovascular research, and recall represents the proportion of known cardiovascular research publications retrieved [7]. Testing was undertaken by two cardiovascular research specialists (MDH, GSB). We amended a previously-used definition of cardiovascular research [5] to develop this filter:

Study of the cardiovascular system (heart and blood vessels) and its functions in health and disease, including heart disease and stroke, invasive/interventional cardiology, cardiac surgery, and vascular surgery, which is the practice of diagnostic and therapeutic procedures that involve entry into the heart and major blood vessels.

With each filter iteration (developed by MDH), the tester (GSB) was given a random sample of 400 titles of publications drawn from the whole retrieval of the cardiovascular filter, mixed with 100 titles of publications from cardiovascular departments. The tester was asked to decide whether titles represented cardiovascular research publications. Iterative amendments were made to the filter. Keywords or headings were included or excluded on the basis of the findings, and tester was given new testing samples. Our initial filter produced precision and recall scores of 0.797 and 0.874, respectively, and after five iterations, our final filter produced precision and recall scores of 0.905 and 0.903.

### Bibliometric Filter Application

We applied our final bibliometric filter to the Thomson Reuters Web of Knowledge Science Citation Index Expanded [SCI-EXPANDED] and Social Sciences Citation Index [SSCI] for publication years 1999–2008 [8]. We searched for all articles, reviews, and conference proceedings as citable items over the study period. After removing duplicates, we merged this output with the corresponding citation report from the Web of Knowledge for each publication to create actual citation indices [ACIs], the average of the running five-year citation count post-publication (total citations five years post-publication divided by five), including the year of publication, to provide comparable citation estimates over time through 2012 [9].

### Bibliometric Filter Analysis

We performed geographical analysis through integer counts and fractional counts based on publication years, as we have previously published [10]. In brief, integer counts give a country a score of one for all authors from that country, which overestimates the number of publications by approximately 20%, depending on publication year and subject [7]. Fractional counts divide the number of publication addresses from an individual country (numerator) by the total number of publication addresses (denominator). For example, comparing a manuscript with one Chinese author and 9 US authors with a manuscript with 9 Chinese authors and one US author, the former example would be a 0.1 fractional count for China and 0.9 for the US, and the latter would be a 0.9 fractional count for China and 0.1 for the US. No weight is assigned by author order or reprint addresses [11]. We estimated international collaboration by the ratio of fractional to integer counts to derive a fractional contribution estimate.

### Analysis by Income, Population, Development, and Burden of Disease

We estimated the relationship between time and cardiovascular publications through linear regression of log-transformed data within the entire dataset by World Bank Income group, including members of the Organization for Economic Cooperation and Development [OECD] [12]. A list of OECD member countries can be found at http://www.oecd.org/general/listofoecdmembercountries-ratificationoftheconventionontheoecd.htm. World Bank income groups include low-income ($1025 or less), lower middle-income ($1026 to $4035), upper middle-income ($4036 to $12 475), and high-income ($12 476 or more) economies based on per capita gross national income. We created replicate models after removing data from the United States from the dataset as a sensitivity analysis (n = 199 vs. 198 countries). We also performed parametric and non-parametric pairwise comparisons on annual cardiovascular publications among World Bank Income groups.

We compared these data against publicly available country-level data, including income status (1999–2008), population size (1999–2008), total number of scientific and technical articles (1999–2008), and Human Development Index (combination of per capita gross domestic product, life expectancy, and education level for years in which data were available, i.e., 2000; 2005–2008) from the World Bank [12] and study mid-point (2004) age-standardized cardiovascular disease morbidity and mortality rates from the Global Burden of Disease [1] to explore their relationships with cardiovascular research output. Novel relationships were explored by creating a Google Motion Chart through *R* v2.15.1 (*R* Foundation for Statistical Consulting; Vienna, Austria) for creation of an interactive website. Other analyses were performed using SAS v9.3 (Cary, NC). Statistical significance of the results was set as two-sided P<0.05.

### Ethics

The Northwestern University institutional review board provided exemption for ethics review since this study did not meet criteria for Human Subject Research. Based on this exemption, no informed consent was obtained.

## Results

**Figure 1:**
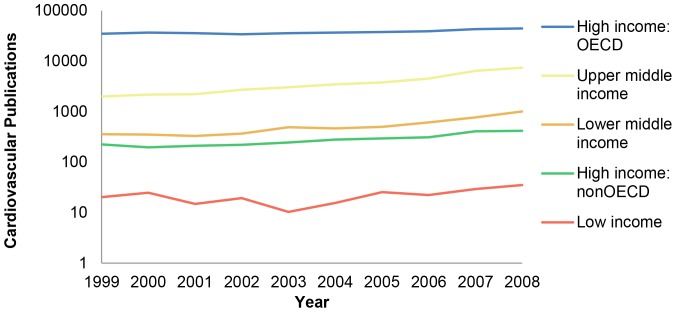
Cardiovascular publications by integer count, by World Bank Income Group (log). Trends in log number of cardiovascular publications by integer count, stratified by World Bank income group (1999–2008).

Table 1 outlines trends in the total number of global cardiovascular research articles downloaded from Web of Knowledge each year, number of citations downloaded, and matched number of records. Cardiovascular integer counts increased from 40,661 publications in 1999 to 55 284 publications in 2008, which represents a 36% increase over that decade. Figure 1 shows these trends on a logarithmic scale stratified by World Bank income. High-income countries that are members of the OECD published more articles in aggregate per year than high-income, non-OECD countries, middle-, or low-income countries. However, the proportion of cardiovascular publications from high-income, OECD countries declined from 93% (1999) of the total share to 84% (2008) of the total share over the study period. The rate of publication increase was greatest in upper-middle-income countries over the study period (336%; driven primarily by China), followed by lower-middle-income countries (291%), high-income, non-OECD countries (246%), low-income countries (73%), and high-income, OECD countries (30%). The majority of this growth appears to be driven primarily by increases in the number of publication outlets (i.e., sources increased by 73% over the study period; data not shown).

**Table 1:** Trends in publications (integer count) and citations downloaded from Web of Knowledge by year using the cardiovascular bibliometric filter.

Year	Publications Downloaded from Web of Knowledge	Publications After Duplicates Removed	Citations Downloaded from Web of Knowledge	Final Matched, Complete Records
1999	40 661	40 661	40 254	37 849
2000	41 603	40 507	41 408	39 876
2001	41 306	40 338	40 171	38 996
2002	41 891	41 792	41 214	37 881
2003	43 490	43 466	42 489	40 034
2004	44 656	44 656	43 676	41 685
2005	46 864	46 863	45 440	43 091
2006	47 929	47 929	47 680	45 257
2007	52 437	52 432	52 437	51 584
2008	55 284	55 284	55 283	54 459
**Total**	**456 121**	**453 928**	**450 052**	**430 712**

Table 2 shows the linear regression results demonstrating the association between time and cardiovascular research publications by income group. Substantial overlap is seen among the effect sizes (beta coefficients) and measures of imprecision (95% CI) by income group despite different baseline levels (intercepts), even after excluding the largest publisher of cardiovascular research as a sensitivity analysis (United States).

**Table 2:** Association between year of publication (per year increment) and cardiovascular publication assessed as a log-transformed outcome.

Country Income Group	Parameter Estimates (β, 95% CI)			
	With US		Without US	
	Intercept	Time (year)	Intercept	Time (year)
High Income: OECD (n* = 31 or 30)	5.85 (5.50, 6.20)	0.07 (0.00, 0.13)	5.73 (5.39, 6.06)	0.07 (0.00, 0.13)
High income: non-OECD (n = 26)	1.20 (0.86, 1.54)	0.05 (−0.01, 0.11)	-	-
Upper middle income (n = 49)	1.93 (1.60, 2.27)	0.09 (0.02, 0.15)	-	-
Lower middle income (n = 49)	0.86 (0.64, 1.07)	0.05 (0.01, 0.09)	-	-
Low income (n = 36)	0.33 (0.20, 0.46)	0.03 (0.01, 0.06)	-	-
All Income Groups (n = 199 or 198)	1.86 (1.66, 2.06)	0.06 (0.02, 0.10)	1.82 (1.63, 2.01)	0.06 (0.02, 0.09)

N represents the number of countries within each income group. Each country contributed 10 years of observations for these regression models.

Table 3 demonstrates trends in the median number of citations per publication by year, stratified by income group and shows a direct gradient by income country status. Median citations per publication increased across all country groups over the study period, and the relative difference between poorer and wealthier countries decreased over time. Median (interquartile range) citations per publication were lowest among publications from low-income countries (0 all years; 0 [0, 0] in 2001) and were highest among publications from high-income, OECD countries (18·4 [12·5, 19·7] in 2007). Lower citation counts in 2008 may reflect incomplete citations reported to Web of Knowledge during follow-up period (2012). Trends were statistically significant for all country groups except upper middle-income countries.

**Table 3:** Trends in median (interquartile range) five year running actual citation index [ACI] counts per publication, stratified by World Bank income group status (1999–2008).

Income Group	1999	2000	2001	2002	2003	2004	2005	2006	2007	2008	Beta coeff. (95% CI)
High income: OECD	11.7	12.9	12.2	14.2	15.6	15.5	16.4	15.8	18.4	16.8	0.13
	(8.3–14.6)	(8.8–15.2)	(9.1–16.3)	(10.1–17.7)	(10.4–18.5)	(11.9–18.2)	(11.8–19.5)	(11.7–18.2)	(12.5–19.7)	(12.1–0.3)	(0.08, 0.18)
High income: non-OECD	2.2	2	0.5	0	2	3.7	3.5	2.9	4.5	3	0.07
	(0–4.1)	(0–5.5)	(0–2.4)	(0–5)	(0–4)	(0–11)	(0–8)	(0–8.8)	(0–8.3)	(0–9.1)	(0.02, 0.13)
Upper middle income	2.5	3.9	3.8	3.7	5.8	4.6	5	5.4	5.3	5	0.05
	(0–6.6)	(0–8)	(0–7.7)	(0–7.9)	(0–9.4)	(0–8.5)	(0–11.1)	(0.5–9.3)	(0.7–10.4)	(0.5–9.1)	(−0.02, 0.11)
Lower middle income	0.3	1.4	0	0	0	0	0	2.3	4	4.3	0.07
	(0–3.3)	(0–4.8)	(0–4.4)	(0–3.8)	(0–3.3)	(0–4.3)	(0–7.1)	(0–6.5)	(0–7.8)	(0–8)	(0.03, 0.11)
Low income	0	0	0	0	0	0	0	0	0	0	0.09
	(0–0.7)	(0–2.3)	(0–0)	(0–1.1)	(0–2)	(0–1)	(0–5.3)	(0–4.5)	(0–7)	(0–7)	(0.01, 0.16)

ACI is calculated by dividing a publication's total citations five years post-publication by five. Lower citation counts in 2008 may reflect incomplete citations reported to Web of Knowledge during follow-up period (2012).

The remaining figures evaluate multiple comparisons through a combination of x and y-axes, color, and bubble sizes to identify novel, country-level relationships. Interactive figures whereby users can manipulate combinations of variables are available at: http://115.115.223.246:8080/ccdcgmc-webapp/generate.ccdcgmc. For the remaining figures in this manuscript, the following format is used: each bubble represents an individual country, and the size of each bubble represents a country's population size. By way of orientation, the largest high-income, OECD country represents the United States, the largest upper-middle-income country represents China, and the largest lower-middle-income country represents India. For continuity, the same color scheme is used for stratification of income groups in all figures.

**Figure 2:**
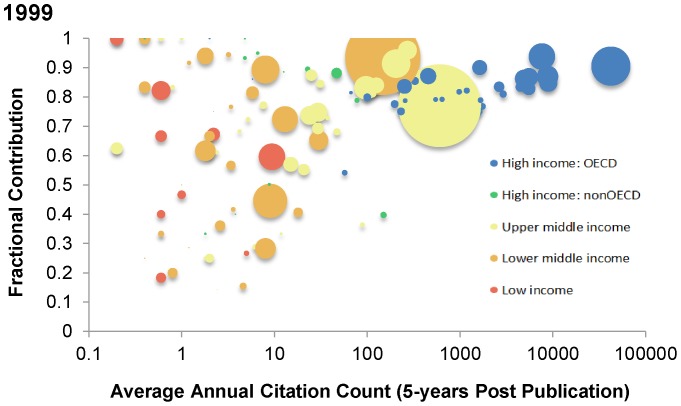
Country-level fractional counts against logarithmic annual citation counts in 1999 (A) and 2008 (B). Country-level fractional counts (scale: 0–1) against logarithmic annual citation counts (country-level cardiovascular research output) with color coding representing World Bank country income status. Fractional counts represent the proportion of each country's authors against the total number of authors on each publication. The size of each country's bubble represents the population size of that country. Average citation index (ACI) is calculated by dividing a publication's total citations five years post-publication by five.

Figures 2A and 2B compare country-level fractional contributions (ratio of fractional to integer counts; scale: 0–1) in 1999 (A) and 2008 (B) against logarithmic annual citation counts (country-level cardiovascular research output) by World Bank country income status to estimate the relative degree of international collaboration. Lower fractional contributions reflect a greater proportion of publications with international collaboration. High-income, OECD countries generally have higher fractional counts than lower income groups, which suggest less international collaboration. There is marked variability in fractional counts among countries with less than 100 annual citations (integer count).

In general, the majority of countries appear to publish a similar proportion of cardiovascular publications against total publications with a modest relative increase over the study period (mean over entire study period = 4·3% of total scientific and technical publications) (Figures S1A and S1B).

**Figure 3:**
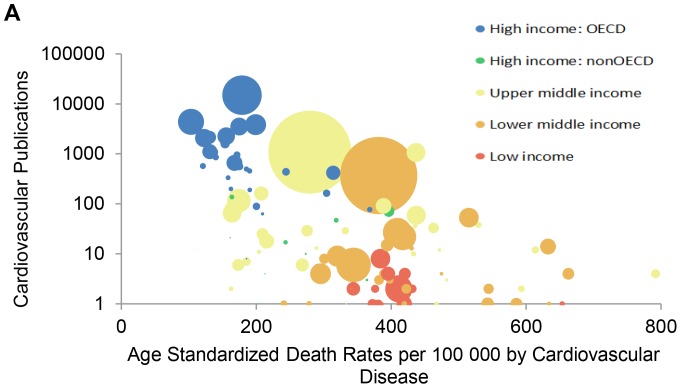
Country-level cardiovascular research publications in 2004 against age standardized death rates per 100 000 (A) and age standardized disability adjusted life year [DALY] rates per 100 000 (B). Color coding representing World Bank country income status. The size of each country's bubble represents the population size of that country.

**Figure 4:**
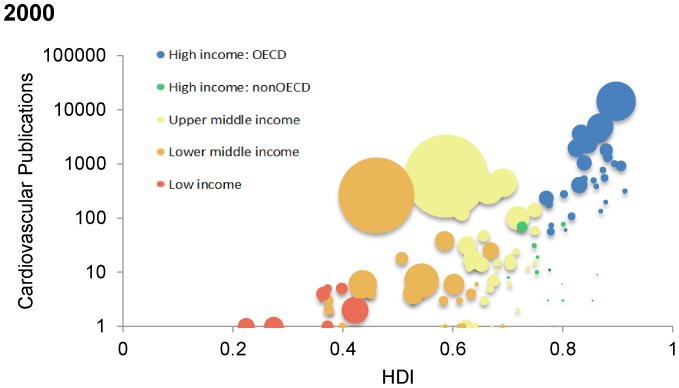
Country-level cardiovascular research publications against Human Development Index in 2000 (A) and 2008 (B). Color coding representing World Bank country income status. The size of each country's bubble represents the population size of that country.

Figures 3A and 3B demonstrate an inverse relationship between integer counts of cardiovascular research publications in 2004 against age-standardized cardiovascular disease mortality per 100 000 (A) and age-standardized cardiovascular morbidity (as measured through disability adjusted life years [DALYs]) (B) derived from the 2004 Global Burden of Disease dataset. Low- and middle-income countries with the higher burdens of cardiovascular disease measured through both deaths and DALYs have lower research output than high-income countries. Lastly, Figures 4A and 4B demonstrate a curvilinear relationship between integer counts of cardiovascular research publications in 2000 (A) and 2008 (B) against the Human Development Index [HDI]. Countries with low HDI scores (generally considered to be <0·5 [12]) demonstrate the lowest numbers of cardiovascular research publications in both 2000 and 2008 despite modest gains over the study period.

## Discussion

### Summary of Results

In this study of global cardiovascular research output from 1999 to 2008, we demonstrate a 36% increase in cardiovascular research output over a ten-year period by applying a bibliometric filter to the Web of Knowledge. High-income, OECD countries have a 9% lower proportion of cardiovascular publications over the study period (93% to 84%). The relative rate of publication growth was highest among upper and lower middle-income countries, and China had the greatest relative increase in cardiovascular research output over the study period. The wider availability of the Internet over the study period for greater international collaboration, access to journals, and ease of electronic submissions has likely contributed to these gains. The mean number of citations per publication has increased across all income groups, but the relative difference between high-income, OECD countries and low-income countries has decreased from a six-fold difference to a 3·5-fold difference between these groups.

Richer countries tend to have more stable, higher fractional contribution estimates, which reflect a lower degree of international collaboration compared with poorer countries. On the other hand, some middle-income countries have had increases in fractional contributions, which may be associated with concomitant economic development. For example, China's fractional contribution has increased from 0·77 in 1999 to 0·85 in 2008, which reflects a higher proportion of Chinese authors of cardiovascular publications, perhaps due to increased cardiovascular research capacity in China. Cardiovascular research output is also directly associated with Human Development Index rather than cardiovascular disease burden, with which it has an inverse association.

### Comparison with Prior Literature

Prabhakaran and colleagues demonstrated a similar distribution of cardiovascular research publications from high-income countries in an analysis of MEDLINE publications in 1994–1995 and 2004–2005 [2]. High-, upper middle-, lower middle-, and low-income countries published 82%, 7%, 7%, and 4% of cardiovascular publications, respectively, although this analysis included only 90 countries. Nevertheless, the ratio of publications by income group was similar between 1994–1995 and 2004–2005. Mendis and colleagues found a similar proportion of cardiovascular research publications from developed market economies in a limited MEDLINE search in 1991 (78%), 1996 (79%), and 2001 (78%) [13]. The differences in these estimates may be due to differences in search strategies and lack of iterative filter testing by Prabhakaran et al. and Mendis et al.

While citations are increasing in the era of reference management software, increases in citations among cardiovascular publications may be a function of larger trends in the topic area under study. Kulkarni and colleagues found in a 2007 analysis of leading general medical journals that cardiovascular publications had, on average, 13 more citations than “other” research articles; however, general medical publications had, on average, nine more citations than “other” articles, suggesting that this reference group may not be particularly useful [14].

McKee and colleagues have suggested several potential reasons why overall research productivity is low among low- and middle-income countries: limited governmental or non-governmental funding, lack of health research strategy, lack of political will to engage globally or to prioritize health research, geographic isolation, and recent or ongoing conflict [15]. Some have argued that the increasing dominance of English language in scientific writing may be one barrier to cardiovascular research growth, particularly among low- and middle-income countries [16]. The recent increase in cardiovascular research output from China, however, appears to dispute this argument.

### Implications

The “10/90” gap outlined by the 1990 Commission on Health Research for Development, which suggests that <10% of the research resources are available for 90% of the world's health problems, appears to be waning modestly for cardiovascular research over the past decade [17]; however, a “16/84 gap” in terms of research productivity remains. The World Health Organization [WHO] has suggested that there is “No Health Without Research” in its planned World Health Report for 2012 in collaboration with *PLoS Medicine*, with an emphasis on health research system strengthening for better healthcare delivery and policymaking [18]. However, publication of this report was delayed for unclear reasons, and the WHO's World Health Report shifted its focus to “the contributions of research to universal health coverage” [19] rather than research capacity building in general. Further, the WHO's recent call to reduce premature (<70 years) mortality from NCDs, including CVD, by 25% by the year 2025 (“25×25 goal”) has limited mention of NCD research capacity building as a means to improve health, although prior calls for enhanced research capacity have been made [20]. The inconsistent emphasis on research capacity building may stall sustained efforts required to develop a research workforce.

Mendis and colleagues highlighted the fact that poor research productivity is both “a consequence and a contributory factor” to the widening gap between cardiovascular morbidity and mortality in wealthier countries compared with poorer countries [13]. We argue that future development assistance for health and health research, such as the recent funding for the Global Alliance for Chronic Diseases (gacd.org) and the National Heart, Lung, and Blood Institute/UnitedHealth-supported Centers of Excellence network (www.nhlbi.nih.gov/about/globalhealth/centers/index.htm), should be expanded to fill this gap in research capacity to help poorer countries, which bear a disproportionate burden of CVD morbidity and mortality, as one tool to improve national cardiovascular health. While delays in discovery to application are common in both high-income and low- and middle-income research settings, the pursuit of research may not be solely evaluated by its potential effect on the burden of disease. In addition, the decision to support increases in health investments should be made in parallel with increases in research investments.

### Strengths/Limitations

Our study has several strengths, including development and iterative testing of a cardiovascular bibliometric filter with high level of precision and recall; long study period with a large number of publications and citations downloaded to evaluate trends over time; and development of an interactive webpage for easy exploration of multivariable relationships capitalizing upon publicly available datasets.

However, our study also has limitations. First, small countries with few cardiovascular research publications had unstable measures of several data points, including fractional count as a measure of international collaboration due to their low sample sizes. These results, in particular, should be interpreted with caution. However, we know of no other method for assessing international research collaboration on a global scale. Second, citations are only one, limited measure of the relative importance of a research article to inform practice and improve care, though they are used widely for comparison across (and within) journals [14], [21]. Third, the exploration of relationships between country income status, size, cardiovascular research publications and measures such as burden of disease and development are susceptible to reverse causality and confounding and should be considered hypothesis-generating. Fourth, we evaluated publications over the study period that were collected through the Web of Knowledge database, which may have excluded publications from other databases, including others that collect relatively more data from LMICs compared with the Web of Knowledge (Latin American and Caribbean Health Sciences Literature [LILACS], e.g.). However, the Web of Knowledge is the largest bibliometric database currently available and provides standardized author address information for analyses like ours.

## Conclusions

Cardiovascular health research has increased substantially in the past decade, with the greatest relative increases in upper middle-income countries such as China. The United States and other high-income, OECD countries remain the dominant producers of cardiovascular research globally, although their share appears to be declining. However, low- and middle-income countries with the higher burdens of cardiovascular disease continue to have lower research output than high-income countries, and thus require targeted research investments to improve cardiovascular health.

## Supporting Information

Figure S1 **Country-level cardiovascular research publications against total scientific and technical publications in 1999 (A) and 2009 (B).** Total scientific and technical publications as reported by the World Bank with color coding representing World Bank country income status. The size of each country's bubble represents the population size of that country.
(TIFF)
