# Supplementary figures and images for: Global Cardiovascular Research Output, Citations, and Collaborations: A Time-Trend, Bibliometric Analysis (1999–2008)
Source: PLoS One. 2013 Dec 31;8(12):e83440. doi: 10.1371/journal.pone.0083440 (PMC3877050; doi:10.1371/journal.pone.0083440)

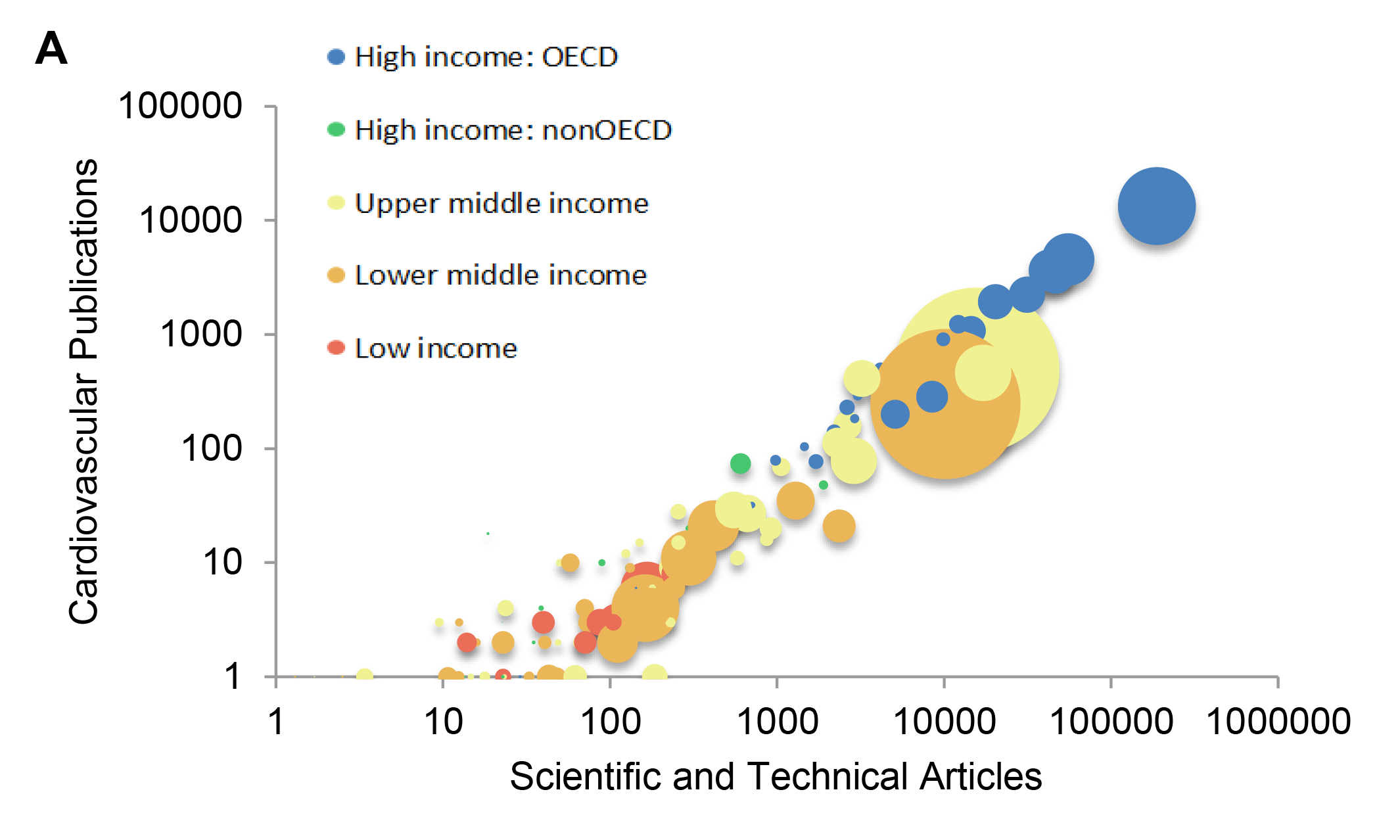

Supplement: Figure S1 — Country-level cardiovascular research publications against total scientific and technical publications in 1999 (A) and 2009 (B). Total scientific and technical publications as reported by the World Bank with color coding representing World Bank country income status. The size of each country's bubble represents the population size of that country. (TIFF) [file pone.0083440.s001.tiff]
